# A New Inhibitor of Tubulin Polymerization Kills Multiple Cancer Cell Types and Reveals p21-Mediated Mechanism Determining Cell Death after Mitotic Catastrophe

**DOI:** 10.3390/cancers12082161

**Published:** 2020-08-04

**Authors:** Mykola Zdioruk, Andrew Want, Anna Mietelska-Porowska, Katarzyna Laskowska-Kaszub, Joanna Wojsiat, Agata Klejman, Ewelina Użarowska, Paulina Koza, Sylwia Olejniczak, Stanislaw Pikul, Witold Konopka, Jakub Golab, Urszula Wojda

**Affiliations:** 1 Laboratory of Preclinical Testing of Higher Standards, Nencki Institute of Experimental Biology, Polish Academy of Science, 02-093 Warsaw, Poland; miko.zdioruk@yahoo.com (M.Z.); a.want@nencki.edu.pl (A.W.); a.mietelska@nencki.edu.pl (A.M.-P.); k.laskowska-kaszub@nencki.edu.pl (K.L.-K.); joanna.wojsiat@gmail.com (J.W.); 2 Laboratory of Animal Models, Nencki Institute of Experimental Biology, Polish Academy of Science, 02-093 Warsaw, Poland; a.klejman@nencki.edu.pl (A.K.); ewelina.uzarowska@warsawgenomics.pl (E.U.); p.koza@nencki.edu.pl (P.K.); w.konopka@nencki.edu.pl (W.K.); 3 OncoArendi Therapeutics, 02-089 Warsaw, Poland; s.olejniczak@oncoarendi.com (S.O.); s.pikul@pikralida.eu (S.P.); 4 Department of Immunology, Medical University of Warsaw, 02-091 Warsaw, Poland; jgolab@wum.edu.pl

**Keywords:** cancer, chemotherapeutic, microtubule-poison, vincristine, mitotic catastrophe, non-apoptotic cell death, p21, p53

## Abstract

Induction of mitotic catastrophe through the disruption of microtubules is an established target in cancer therapy. However, the molecular mechanisms determining the mitotic catastrophe and the following apoptotic or non-apoptotic cell death remain poorly understood. Moreover, many existing drugs targeting tubulin, such as vincristine, have reduced efficacy, resulting from poor solubility in physiological conditions. Here, we introduce a novel small molecule 2-aminoimidazoline derivative—OAT-449, a synthetic water-soluble tubulin inhibitor. OAT-449 in a concentration range from 6 to 30 nM causes cell death of eight different cancer cell lines in vitro, and significantly inhibits tumor development in such xenograft models as HT-29 (colorectal adenocarcinoma) and SK-N-MC (neuroepithelioma) in vivo. Mechanistic studies showed that OAT-449, like vincristine, inhibited tubulin polymerization and induced profound multi-nucleation and mitotic catastrophe in cancer cells. HeLa and HT-29 cells within 24 h of treatment arrested in G2/M cell cycle phase, presenting mitotic catastrophe features, and 24 h later died by non-apoptotic cell death. In HT-29 cells, both agents altered phosphorylation status of Cdk1 and of spindle assembly checkpoint proteins NuMa and Aurora B, while G2/M arrest and apoptosis blocking was consistent with p53-independent accumulation in the nucleus and largely in the cytoplasm of p21/waf1/cip1, a key determinant of cell fate programs. This is the first common mechanism for the two microtubule-dissociating agents, vincristine and OAT-449, determining the cell death pathway following mitotic catastrophe demonstrated in HT-29 cells.

## 1. Introduction

As cancer becomes ever more ubiquitous, affecting a significant proportion of people in developed nations and increasing numbers in developing nations, there is a pressing need for a greater diversity of effective and well-tolerated treatments. Uncontrolled cell division, the hallmark of all cancers, results from deficient control of the cell cycle and from impaired cell death signaling. The cell cycle consists of four distinct phases (G1, S, G2 and M) that are demarcated by checkpoints at G1/S, intra S, G2/M and spindle assembly checkpoint in the M phase. At these checkpoints, cell cycle progression can be stalled whilst defects are identified and the defects either repaired or, in the case of repair failure, one of the non-lethal or lethal mechanisms protecting the cell from damage and malignant transformation are activated [1,2]. In particular, damage in mitosis (M) can activate mitotic catastrophe, an oncosupressive mechanism of mitotic arrest characterized by premature chromosome condensation, chromosomal breaks and micronucleation as well as deficient karyokinesis resulting in the formation of large, multi-nucleated cells. Mitotic catastrophe is a non-lethal process that can eventually result in cellular senescence, apoptosis or non-apoptotic cell death, but the underlying mechanisms governing any resultant cell death pathway are unclear (reviewed in [2,3,4]).

The cell cycle checkpoints are often subverted in cancerous cells, and failed mitotic catastrophe constitutes one of the major gateways to the development of cancer. In turn, the induction of mitotic catastrophe is considered to offer significant advantages in the development of existing and novel anticancer therapies. These advantages include: (a) particular sensitivity of cancer cells to mitotic catastrophe due to their genomic instability associated with polyploidy/aneuploidy; (b) significantly lower doses of cytotoxic agents required for the induction of mitotic catastrophe compared to doses required for direct activation of apoptosis/necrosis, which could limit side effects; (c) the possibility to overcome chemoresistance, which in various cancers is caused by a blockage of mitotic catastrophe (reviewed in [2,3,5]).

Existing chemotherapeutics that induce mitotic catastrophe can be classified, based on mechanism of action, as agents which damage DNA or agents which target microtubules. The latter group comprises such compounds as nocodazole, paclitaxel and vincristine [6,7,8], which interfere with the dynamics of microtubule biochemistry by either promoting microtubule polymerization (Taxans) or depolymerization (Vinca alkaloids). Impairments in microtubule organization lead to failures in such vital processes as chromatids connecting to kinetochores prior to cell division and activation of one or both of the G2/M and M checkpoints [9]. Apart from the arrest of the cell cycle, microtubule poisons such as vincristine have also been shown to cause mitotic catastrophe and impair apoptosis [10], but the molecular mechanisms underlying these cellular steps and their interconnections in cancer cells remain largely unknown.

As pharmaceutical molecules, many routinely-used microtubule poisons are characterized by poor solubility, which necessitates higher doses and increases the likelihood of off-target effects. Noscapine, originally identified as an antitussive, has been shown to exert antitumor effects with fewer side-effects than other molecules that act on microtubules [11]. In spite of this, noscapine and other drugs of its class are not currently listed as recommended first-line therapies, indicating yet more potential for new drugs with similar properties [12,13].

In this work, we have examined the effects of a novel class of 1-(substituted-sulfonyl)-2-aminoimidazoline derived, synthetic, small molecules [14] on a range of cancer cell lines in vitro, and in vivo, in two tumor xenograft mouse models. The chemical structure of these compounds provides easy water solubility, suggestive of the potential for enhanced bioavailability in vivo [15,16], and so represents a possible way to augment the current cancer treatment landscape. We found that OAT-449 (molecular weight 232 Da; for comparison, vincristine’s MW is 825 Da), one of these new, small, water-soluble 2-aminoimidazoline derivatives, is a potent inhibitor of tubulin polymerization, which at nanomolar concentrations induces mitotic catastrophe in colorectal adenocarcinoma HT-29 and cervical adenocarcinoma HeLa cells. Moreover, this study reveals a common mechanism for both OAT-449 and vincristine driving microtubule depolymerization-induced mitotic catastrophe to non-apoptotic cell death in HT-29 cells. This mechanism involves p53-independent cytoplasmic accumulation of p21/waf1/cip1 protein, one of the key cell fate determinants with the ability to inhibit apoptosis when located in the cytoplasm.

## 2. Materials and Methods

### 2.1. Reagents

OAT-449 compound was synthesized and supplied by OncoArendi Therapeutics [14]. For all in vitro experiments, 1 mM of OAT-449 stock solution was prepared in DMSO and stored light-protected at −20 °C. The final concentration of DMSO in the experiments was 0.1%. For in vivo experiments, OAT-449 was dissolved in solutol (Koliphor HS 15, Sigma-Aldrich (St. Louis, MO, USA)): in 30% solutol for HT-29 and in 10% solutol for SK-N-MC xenotransplant mouse models. As a vehicle control, 30% or 10% solutol was used, respectively. Vincristine was purchased from Trimen Chemicals (Lodz, Poland) and CPT-11 (irinotecan) from Sigma-Aldrich. All other chemicals were purchased from Sigma-Aldrich, unless otherwise stated.

### 2.2. Antibodies

Anti-phospho-histone H3 (Ser10), anti-phopsho-p53 (Ser15), anti-p53, anti-p21, Waf/Cip1 (12D1), anti-phospho-NuMA (Ser395), anti-phospho-CDK1 (Tyr15), anti-β-tubulin, anti-cleaved caspase-3, anti-phospho-Aurora B (Thr232), anti-β-actin antibodies and fluorescent dye Draq 5 were purchased from Cell Signaling Technology (Danvers, MA, USA). Anti-Lamin B antibody was obtained from Santa Cruz Biotechnology (Santa Cruz, CA, USA). Anti-PARP antibody was acquired from BD Bioscience (San Jose, CA, USA).

### 2.3. Cell Lines

Eight human cancer cell lines—colorectal adenocarcinoma HT-29, cervical adenocarcinoma HeLa, prostate carcinoma DU-145, epithelioid pancreatic carcinoma (Panc-1), neuroepithelioma SK-N-MC, ovary adenocarcinoma SK-OV-3, breast adenocarcinoma MCF-7, and lung carcinoma A-549—were freshly purchased from the American Type Culture Collection (ATCC) (Manassas, VA, USA) and cultured according to the ATCC recommendations. Cells were cultured in recommended media supplemented with 10% (v/v) fetal bovine serum (FBS), 10 μg/mL streptomycin and 100 U/mL penicillin at 37 °C in a humidified atmosphere containing 5% CO_2_.

### 2.4. Experiments in Tumor Xenograft Mouse Models

BALB/c Nude immunodeficient male mice (unable to produce T lymphocytes) were obtained from Janvier labs (France). Ethical permission was granted by Local Ethical Committee of the Warsaw University of Life Sciences in Warsaw, reference: 432/2013. At the beginning of the experiment, mice were 3 months old. During the experiment, the animals were bred in separate, individually ventilated cages (IVC), with ad libitum access to food and water. The animals were injected subcutaneously around the neck with the mixture of tumor cells and matrigel (BD Biosciences) (1:1) with PBS (Gibco) in a final volume of 100 µL. A preliminary experiment was performed in which the optimal number of cancer cells administered giving linear tumor growth was selected [17]. After optimization, the BALB/c Nude mice xenografts were injected with 3.5 × 10^6^ HT-29 cells or with 7 × 10^6^ SK-N-MC cells. When the tumor reached 100 mm^3^ in volume, administration of the tested substances was started. In mice xenografted with HT-29 cells, OAT-449 was administered intraperitoneally at a dose of 5 mg/kg in 30% solutol, every day for 5 days, followed by 2 day intervals. In the same way, 30% solutol alone (vehicle) was administered as a negative control. As the positive control irinotecan (CPT-11) dissolved in 10% solutol, a final concentration of 3 mg/mL was used and administered intraperitoneally every 3 days. In mice xenografted with SK-N-MC cells, OAT-449 in 10% solutol was administered intravenously at a dose of 2.5 mg/mL, as well as vincristine (1 mg/kg every 7 days) and diluent (10% solutol, every 5 days). Measurements of tumor volume were made every 3 days using an electronic display caliper. Two dimensions were measured: length (L) for the longest dimension of a tumor and width (W) as the dimension perpendicular to the length. Tumor volume was calculated according to the formula: V = (L × W2)/2 (mm^3^).

### 2.5. In Vitro Tubulin Polymerization Assay

The fluorescence-based tubulin polymerization assay was conducted using the Tubulin Polymerization Assay Kit from Cytoskeleton (Cat. #BK011P; Cytoskeleton, Denver, CO) following the manufacturer’s protocol. The reaction was performed in a final volume of 10 μL of PEM buffer (80 mM PIPES, 0.5 mM EGTA, 2 mM MgCl_2_, pH 6.9) containing 2 mg/mL bovine brain tubulin, 10 μM fluorescent reporter, and 1 mM GTP in a 96 well plate at 37 °C. OAT-449 was added to 3 μM final concentration, and 500 μM CaCl_2_ or 3 μM paclitaxel were tested as positive and negative controls for the inhibition or enhancement of tubulin polymerization, respectively. Tubulin polymerization was determined by measuring the fluorescence emission at λ = 420 nm (excitation λ = 360 nm) for 1 h at 1 min intervals using a Flexstation 3 microplate reader (Molecular Device LLC, Sunnyvale, CA, USA). The data were analyzed using Soft Max Pro 4.4.1. software (Molecular Device LLC, Sunnyvale, CA, USA).

### 2.6. Whole Cells Analysis of Tubulin Polymerization

HT-29 cells were plated at 1 × 10^5^ cells/mL in 1 mL medium and allowed to adhere in 12-well plates for a minimum of 12 h. Compounds (OAT-449, paclitaxel or vincristine) were then added at a concentration of 100 nM in DMSO stocks (0.1% DMSO final). After 18 h incubation, the cells were transferred to 2 mL tubes and pelleted (600 g, 3 min). The medium was removed by aspiration 15 min prior to the indicated time point, and cells were trypsinized for 15 min with 0.2 mL of 0.5% trypsin with EDTA prior to pelleting. The medium was aspirated, and the cells fixed by adding 1 mL of 0.5% glutaraldehyde in microtubule stabilizing buffer (MTSB: 80 mM Pipes, 1 mM MgCl_2_, 5 mM EDTA, and 0.5% Triton-X-100, pH 6.8). Following a 10 min incubation at room temperature, the glutaraldehyde was quenched by the addition of 0.7 mL of 1 mg/mL NaBH_4_ in PBS. The cells were pelleted (1000 g, 7 min), and the supernatant removed by gentle aspiration. Next, the cells were resuspended in 20 μL of 50 μg/mL RNase A in the antibody diluting solution (PBS, 0.2% Triton-X-100, 2% bovine serum albumin, and 0.1% NaN_3_, pH 7.4) and incubated overnight at 4 °C. Prior to analysis, 5 μL of anti-β-tubulin–FITC antibody was added to each sample to achieve a final dilution of 1:250, and the cells were incubated in the dark for 3 h. Next, 200 μL of 50 μg/mL propidium iodide (PI) in PBS was added, and cells were transferred to polyester tubes, vortexed, equilibrated to room temperature, and analyzed by flow cytometry using a FACSCalibur instrument (Becton Dickinson, Germany). Whole cells were identified based on their forward scatter and side scatter signals and PI staining (single cells based on pulse width and height). Exactly 10,000 events from each sample were collected in a single cell gate. Data were analyzed using BD CellQuest Pro Software.

### 2.7. Cell Viability 3-(4,5-Dimethylthiazol-2-yl)-2,5-diphenyltetrazolium Bromide MTT Assay

Cells were seeded at 1 × 10^4^ cells/well in 96-well plates. After 72 h of treatment at various concentrations of OAT-449 or vincristine, cells were incubated with MTT at a final concentration of 1 mg/mL for 3 h at 37 °C. Then, the culture medium was removed, and the purple formazan crystals were dissolved with DMSO. The extent of MTT reduction was quantified based on the absorbance measurements at 595 nm with 630 nm as a reference wavelength, using a microplate reader (iMark, Bio Rad, CA, USA). Relative EC_50_ values were calculated by nonlinear regression curve fitting lines using GraphPad Prism 4 Software (GraphPad Software, San Diego, CA, USA).

### 2.8. Preparation of Whole-Cell Extracts and Subcellular Fractionation

To prepare whole-cell lysates, cells were harvested, washed in PBS, and then lysed in ice-cold lysis buffer (50 mM Tris, 150 mM NaCl, 50 mM NaF, 1% Nonidet P-40, pH 7.4), containing 1 mM sodium orthovanadate, 1 mM PMSF, 1 mM sodium pyrophosphate, and protease inhibitor Complete Mini Mixture (Roche).

To separate the cytosolic and nuclear fractions, cells were harvested, washed in PBS, and then lysed in ice-cold hypotonic buffer (10 mM HEPES, 10 mM KCl, 0.1 mM EDTA, 0.1 mM EGTA, 1 mM sodium orthovanadate, 1 mM sodium pyrophosphate, 1 mM PMSF, and protease inhibitor mixture, pH 7.9). After extraction on ice for 15 min, 0.5% Nonidet P-40 was added and the lysed cells were centrifuged at 700 *g* for 10 min. Supernatants containing cytosolic proteins were separated, and pelleted nuclei were washed twice with the hypotonic buffer, and then lysed in the hypertonic buffer (20 mM HEPES, 0.4 M NaCl, 1 mM EDTA, 1 mM EGTA, 1 mM sodium orthovanadate, 1 mM sodium pyrophosphate, 1 mM PMSF, and protease inhibitor mixture, pH 7.9). After extraction on ice for 30 min, the samples were centrifuged at 10,000 *g* for 15 min at 4 °C. Antibodies to β-actin and to lamin B were used to assess the purity of the cytosolic and nuclear fractions, respectively. The protein concentration in the extracts was determined by the BCA Protein Assay Kit (Pierce, Rockford, USA).

### 2.9. Immunoblotting

Proteins were separated by sodium dodecyl sulfate-polyacrylamide gel electrophoresis (SDS-PAGE) and transferred to a PVDF membrane (Immobilon-P, Bio-Rad Laboratories, Richmond, CA, USA). Equal amounts of protein (20–50 µg) were loaded in each lane. Uniformity of sample loading and transfer integrity were verified by staining with Ponceau-S. After transfer, the PVDF membranes were blocked with nonfat milk and incubated overnight at 4 °C with primary antibodies followed by secondary antibodies conjugated to horseradish peroxidase (HRP). The levels of β-tubulin as a reference were detected using polyclonal rabbit anti-β-tubulin antibody, tubulin having already been widely used in similar experiments with microtubule poisons [18,19,20]. Detection of the immunoreaction was performed with an enhanced chemiluminescence (ECL) kit (Amersham BioSciences, Amersham, UK). Protein band densities were quantified using ImageJ software (NIH, Bethesda, MD, USA) after scanning the images with a ChemiDoc MP Imaging System (Bio-Rad).

### 2.10. Cell Cycle Analysis by DNA Content Measurement

Flow cytometry was used to analyze distribution of cells in the SubG1, G1, S and G2/M cell cycle phases based on PI staining. Cells were seeded at an initial concentration of 1 × 10^6^ cells/mL in the absence or presence of 30 nM OAT-449 or vincristine. Untreated and treated cells were next washed with PBS, suspended in 70% cold ethanol for 1 h, washed again and incubated for 1 h in 0.1% sodium citrate in PBS containing RNase A (10 µg/mL) and 50 µg/mL PI. Prior to the analysis, the cells were equilibrated to room temperature and then analyzed using the FACSCalibur instrument (Becton Dickinson, Germany). Exactly 10,000 events from each sample were collected in a single cell gate. Data were analyzed using BD CellQuest Pro Software.

### 2.11. Annexin V Flow Cytometry Apoptotic Assay

Apoptosis was measured by flow cytometry using a MUSE Annexin V kit (Merck, Germany). Cells were seeded at an initial concentration of 1 × 10^6^ cells/mL in the absence or presence of OAT-449 or vincristine. After 24 h, cells were collected, centrifuged (400 *g*, 4 °C, 5 min), and washed with 1 mL of PBS. Afterward, cells were incubated for 15 min in the dark on ice in 100 μL of the Annexin V incubation buffer (0.1 M HEPES, 25 mM CaCl_2_, 1.4 M NaCl, pH 7.4), with 1 µL of Annexin V-FITC. After incubation, 400 μL of the Annexin binding buffer and 2 μL of PI (50 μg/mL) were added. Measurements were performed using a MUSE analyzer (Merck, Germany) and data were analyzed using MUSE Analysis 14.1 software.

### 2.12. Immunocytochemistry and Confocal Microscopy of Tubulin Protein

HT-29 and HeLa cells were cultured on coverslips and were treated with 0.1% DMSO alone and either with 30 nM OAT-449 or 30 nM vincristine in 0.1% DMSO, in triplicate. After 24 h of treatment, the cells were fixed with a PEM buffer (80 mM PIPES, 1 mM MgCl_2_, 1 mM EGTA, 3% sucrose, 0.1% glutaraldehyde, 4% formaldehyde, pH 6.8) for 15 min and permeabilized with 0.5% Triton-X-100 for another 15 min at room temperature. The staining was performed using an anti-β-tubulin antibody from Cell Signaling Technology (Danvers, MA, USA) according to the manufacturer’s protocol. The cells were incubated with the anti-β-tubulin antibody overnight at 4 °C and, after washing, with a secondary antibody conjugated to Alexa Fluor 488. The staining results were observed under a confocal microscope Leica TCP SP8. Obtained images were analyzed using Leica Application Suite X 1.1.0.12420 software.

### 2.13. Statistics

All of the in vitro experiments were performed in biological triplicate. The summary statistics used were as described in the accompanying figure legends, representative of at least three independent experiments. Statistical analysis was performed with GraphPad Prism (GraphPad, San Diego, CA, USA).

The in vivo experiments were performed on 7–11 animals per group: in the HT-29 xenograft model, each group consisted of 11 mice. In the SK-N-MC xenograft model, each group consisted of 7–8 mice; in the group treated with OAT-449, the experiment was successfully conducted in 4 mice, while in the remaining 3 animals the experiment was inconclusive due to atypical tumor presentation. All results are presented as means ± SEM. Comparisons between groups were analyzed using two-way ANOVA. Probability values of *p* < 0.05 were considered to be statistically significant. Where statistically significant effects were detected using ANOVA, a post hoc Newman–Keuls test was applied to determine differences between groups.

## 3. Results

### 3.1. OAT-449, in Micromolar Concentrations, Kills a Range of Cancer Cell Lines with Similar Efficacy to Vincristine

**Figure 1:**
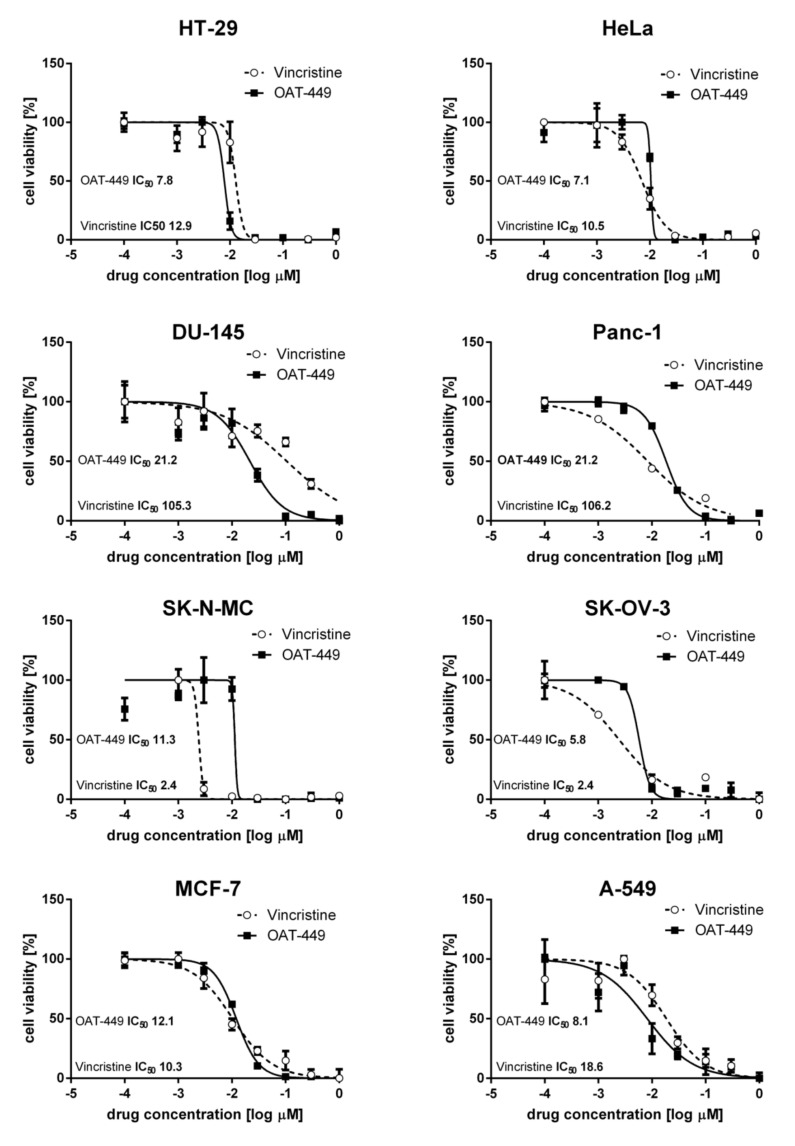
OAT-449, similarly to vincristine, strongly inhibits viability in multiple cancer cell lines. Live cells were measured by 3-(4,5-dimethylthiazol-2-yl)-2,5-diphenyltetrazolium bromide (MTT) assay, in which 10^4^ cells were seeded in 96-well plates treated with OAT-449, vincristine or 0.1% DMSO as control and analyzed 72 h after treatment. Log(dose)–response curves for HT-29, HeLa, DU-145, Panc-1, SK-N-MC, SK-OV-3, MCF-7 and A-549 are shown as labeled. Each data point represents the mean ± SEM of at least 3 independent experiments. Each EC_50_ was calculated based on sigmoidal curve fitting to the respective data set.

From an in vitro cytotoxicity pre-screen of 20 novel synthetic OAT compounds based on a common backbone [14], we selected OAT-449 as a good candidate molecule for further investigation in a multimodel study. Cystostatic/cytotoxic effects of OAT-449 were measured in eight different cancer cell lines 72 h after treatment, and these effects are displayed in Figure 1, with vincristine being used as a direct comparator. In the eight cell lines tested, there is an expected degree of variation, where one or other treatment has a larger effect on particular cell lines. For example, vincristine is an order of magnitude better at killing SK-N-MC cells than OAT-449, while OAT-449 is an order of magnitude better at eliminating DU-145 and Panc-1 cells. Moreover, the EC_50_ of our compound does not rise above 21.2 nM, suggesting a broad efficacy for all cases, adequate if not optimal for therapeutic use.

### 3.2. OAT-449 Inhibits Human Tumor Growth in Two Xenograft Mouse Models

**Figure 2:**
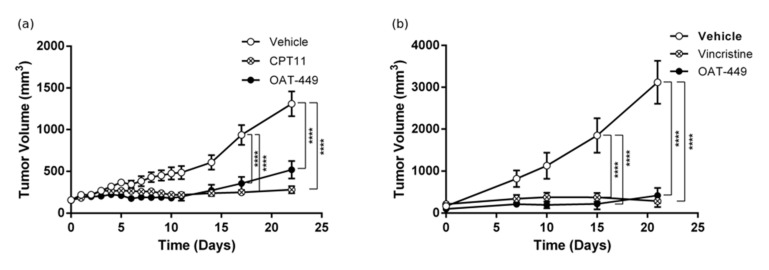
OAT-449 inhibits tumor growth in HT-29 and SK-N-MC xenografts into BALB/c Nude mice. (**a**) Mean tumor volume of HT-29 cell xenografts, measured every 3 days during drug administration. OAT-449 and vehicle solutol (5 following days, 2 day intervals), CPT-11 (40 mg/kg q3d) were administered IP. The number of animals in each group was 11. (**b**) Mean tumor volume in mice xenografted with SK-N-MC cells during drug administration. Tumor size was measured every 3 days. OAT-449 was administered intravenously at a dose of 2.5 mg/mL, as well as vincristine (1 mg/kg every 7 days) and diluent (solutol, every 5 days). *N* = 8 for vehicle and vincristine group and *n* = 4 for OAT-449 group. **** *p* < 0.0001.

In the next step, we tested OAT-449 anti-tumor activity in two xenografts of human tumor cell lines in the BALB/c Nude mouse model (Figure 2). For xenotransplantation, two cell lines were selected from those which demonstrated sensitivity to OAT-449 in our in vitro assays shown in Figure 1: colorectal adenocarcinoma HT-29 and neuroepithelioma SK-N-MC. Mice xenografted with HT-29 cells (Figure 2a), and OAT-449 administered intraperitoneally at a dose of 5 mg/kg, daily for 5 days followed by 2 day intervals thereafter, showed a significant antitumor effect compared with the negative control (vehicle, solutol alone, (Sigma-Aldrich, St Louis, MO, USA)). The tumor growth inhibition rate was similar to that observed for irinotecan (CPT-11) employed as the efficient positive control for colorectal cancer cell lines [21]. For the next xenograft experiment, we selected SK-N-MC cells, a human tumor cell line with well-documented sensitivity to vincristine [19]. In this model, vincristine as a positive control was administered intravenously at a dose of 1 mg/kg every 7 days (Figure 2b). Accordingly, we tested lower doses and administration frequencies of OAT-449 than in the first xenograft model. OAT-449 was administered intravenously at a dose of 2.5 mg/kg, every 5 days, similar to the vehicle control (solutol). OAT-449 demonstrated tumor growth inhibition similar to vincristine (Figure 2b). In summary, these data encourage further preclinical evaluation of OAT-449, including determining OAT-449 doses and other parameters of administration in various tumor models, required for defining a potential therapeutic window.

### 3.3. OAT-449 Exerts Its Cytotoxicity through Inhibition of Tubulin Polymerization In Vitro and in Cells

**Figure 3:**
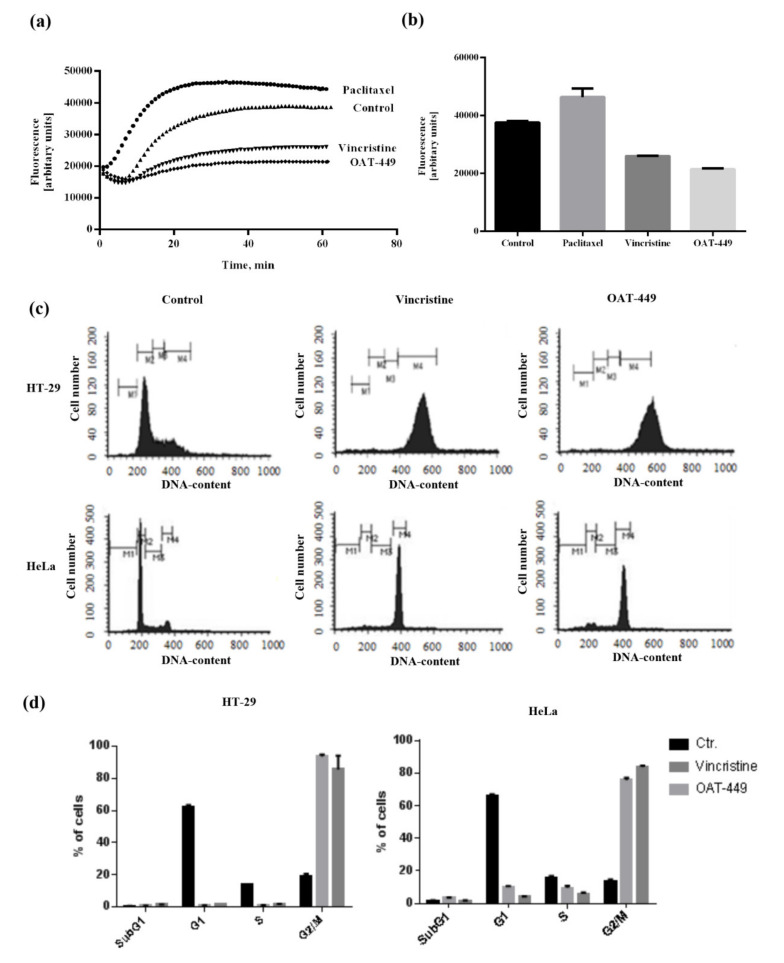
Both OAT-449 and vincristine inhibit tubulin polymerization and cell cycle progression. (**a**) In vitro polymerization activity of fluorescently labelled tubulin was monitored either in the absence (control) or following the addition of OAT-449 (3 μM) or paclitaxel (3 μM) (negative control) or vincristine (3 μM) (positive control). (**b**) Graph represents a maximum value for each sample, mean ± SEM of at least 3 independent experiments. (**c**) Representative cell cycle profiles of HT-29 and HeLa cells treated with OAT-449 (30 nM), vincristine (30 nM) or DMSO (0.1%) as control, stained after 24 h with propidium iodide and analyzed by flow cytometry. Data shown are taken from 3 independent experiments. (**d**) Mean ± SEM of proportion of cells in different cell cycle stages, under specified treatment conditions.

Using a purified, fluorescently-labelled, tubulin assay, we demonstrate in Figure 3a,b that OAT-449 prevents the polymerization of tubulin in a manner similar to vincristine, and in direct contrast to paclitaxel—a known microtubule stabilizer. OAT-449 is, in fact, even more effective at destabilizing tubulin polymers than vincristine. This destabilizing effect of OAT-449 on tubulin was also recapitulated in cultured colorectal adenocarcinoma HT-29 cells, as shown in Supplementary Figure S1, measured in whole cells by flow cytometry.

### 3.4. In HT-29 and HeLa Cells, Both OAT-449 and Vincristine Results in G2/M Arrest but Not in Apoptosis

**Figure 4:**
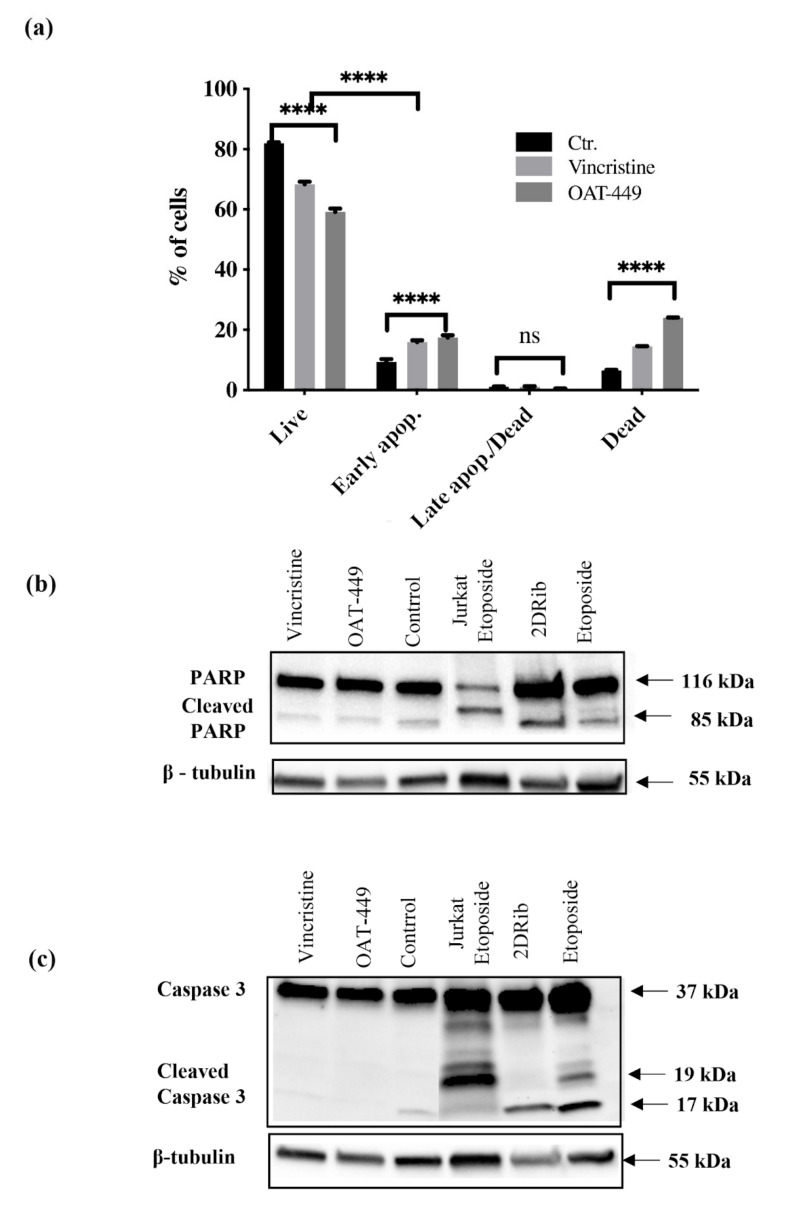
Lack of apoptotic response of HT-29 cells after treatment with OAT-449 or vincristine. (**a**) Cells were incubated for 18 h at initial density of 1 × 10^6^ cells/mL in the absence or presence of 30 nM OAT-449 or vincristine. After treatment cells were stained with Annexin V-FITC. Data represent mean ± SEM of at least 3 independent experiments (**** *p* value < 0.0001). (**b**) Immunoblotting of PARP in HT-29 cells in basal conditions and upon treatment. (**c**) Immunoblotting of pro-apoptotic caspase-3 in whole cell lysate of HT-29 in basal conditions and upon treatment with OAT-449 or vincristine. 2-deoxy-D-ribose (2DRib) and etoposide were used as a positive controls in order to achieve activation of apoptotic response driven via cleavage of poly (ADP-ribose) polymerase (PARP) and caspase-3. β–tubulin used as a loading control. Blots are representative of 3 independent experiments.

As visible in Figure 3c, after 24 h, HeLa and HT-29 cancer cell lines exhibit similar responses to treatment with vincristine or OAT-449, in the majority of the cells, in each case displaying 2n DNA content consistent with G2/M arrest. It is also notable that the arrested G2/M peak for HT-29 cells is markedly higher than the 2n position (~450 on the DNA content scale), which could indicate multi-nucleation. We also noted small subG1 peaks associated with OAT-449 treatment, which is consistent with data in Figure 4, showing only a very small degree of apoptosis measured by Annexin V and PI (Figure 4a). Furthermore, Western blots for PARP (Figure 4b) and caspase-3 (Figure 4c) demonstrate no sign of activation of apoptotic execution machinery at the protein level. Thus, upon OAT-449 and vincristine treatment at the applied concentrations within 24 h, the cells respond with G2/M arrest and inhibition of canonical apoptotic mechanisms. Later, 72 h after treatment, cell debris was observed in the absence of apoptotic cells, in agreement with the necrotic cell death scenario [22].

### 3.5. Both OAT-449 and Vincristine Generates Mitotic Catastrophe in Cancer Cells

**Figure 5:**
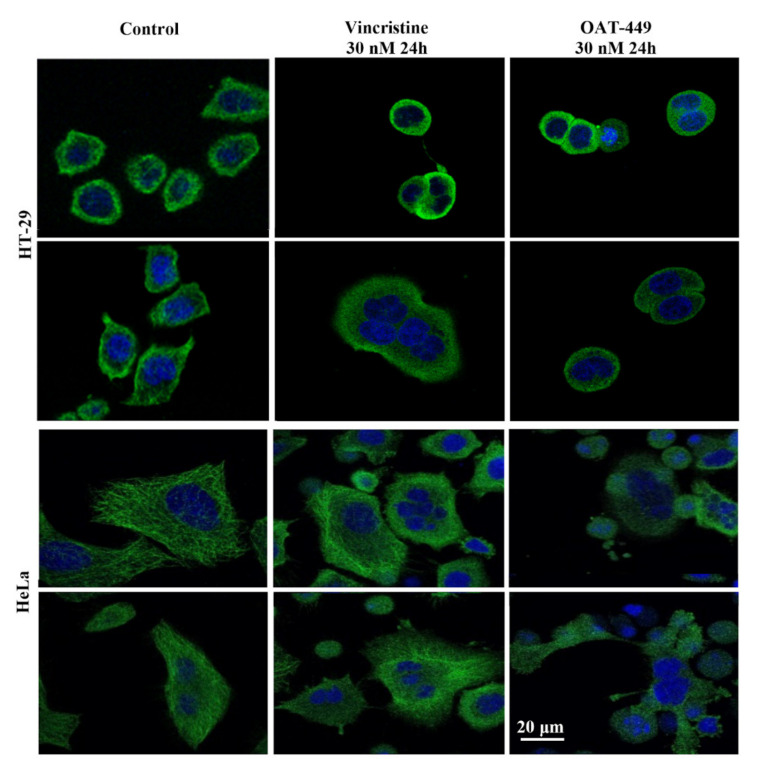
Both OAT-449 and vincristine cause mitotic defects, multi-nucleation, aneuploidy and polyploidy through the blocking of the tubulin polymerization and induces G2/M phase cell cycle arrest. Cancer cells HT-29 and HeLa were grown on coverslips and treated with control culture medium, 30 nM OAT-449 or vincristine for 24 h, fixed with 2% formaldehyde, stained with DAPI and Alexa Fluor 488-conjugated anti-tubulin antibody (green fluorescence) and examined using confocal microscopy. The images are representative of at least 3 independent experiments.

Congruent with the cell cycle data, we directly visualized cells 24 h after treatment with OAT-449 or vincristine, and the resulting multi-nucleated and improperly divided HT-29 and HeLa cells can be seen in Figure 5. More visible with HeLa cells, due to their larger cytoplasmic area, is the breakdown of the fine structure of tubulin within the cell first with exposure to vincristine, then compared with OAT-449 treatment. This is consistent with earlier observations regarding the ex vivo and in vitro inhibition of tubulin polymerization in Figure 3 and Figure S1. These data provide clear evidence of induction of mitotic catastrophe by OAT-449, similarly with vincristine, in cancer cells.

### 3.6. Both OAT-449 and Vincristine Cause Changes in Phosphorylation Status of Cell-Cycle Regulatory Proteins in HT-29 Cells

**Figure 6:**
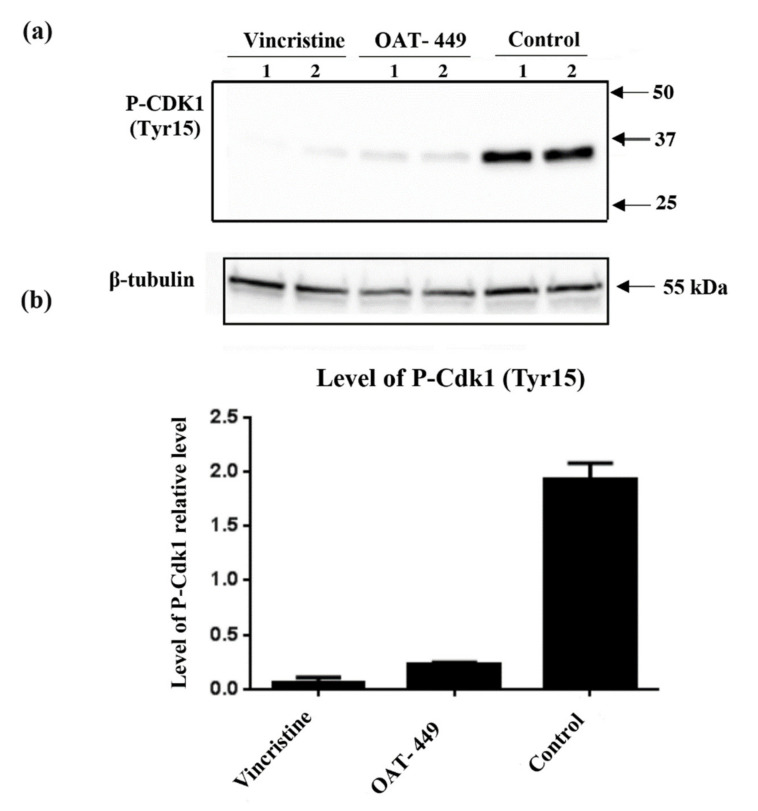
OAT-449 causes a reduction in phosphorylation of Cdk1 immunoblotting of P-Cdk1 (Tyr15) in whole cell lysate of HT-29 in basal condition and upon 18 h treatment with 30 nM OAT-449 or vincristine. (**a**) Immunoblot, showing biological duplicate lanes (1–2) for each condition. (**b**) Densitometry of immunoblot data, with signal intensity relative to β-tubulin (mean ± SEM). Data are representative of 3 independent experiments.

**Figure 7:**
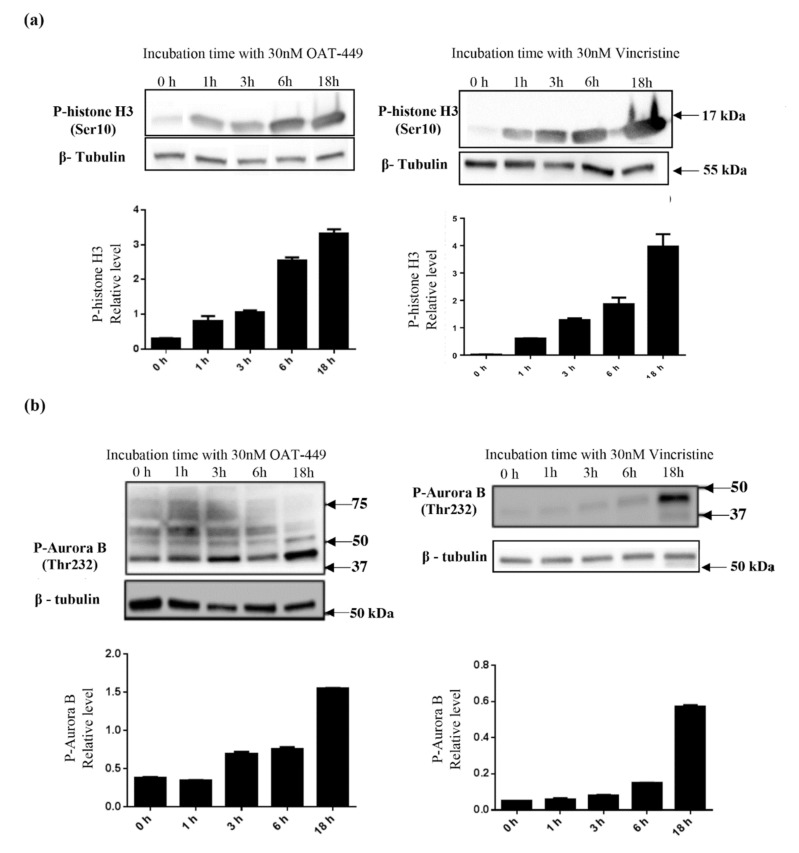

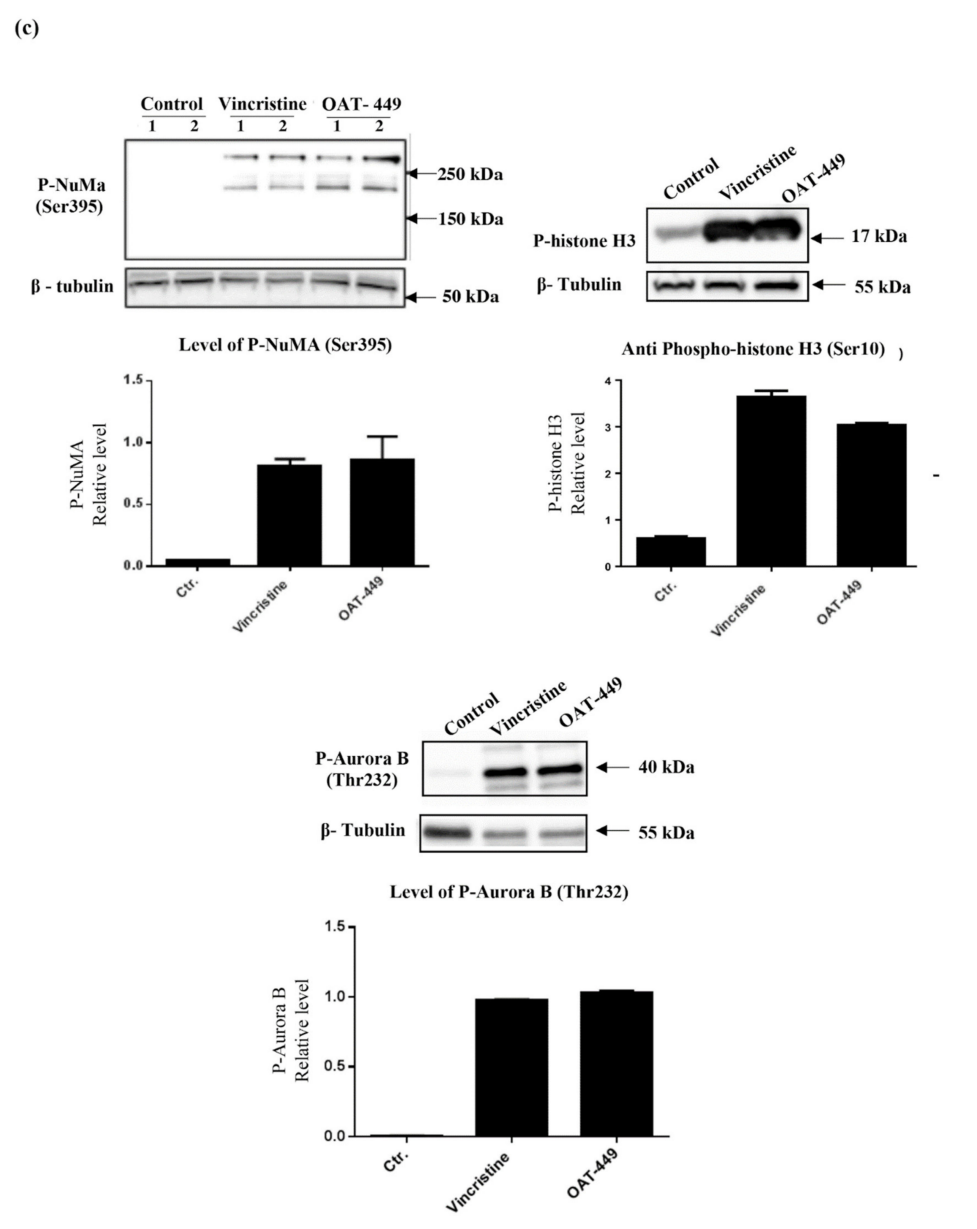
Both OAT-449 and vincristine modify phosphorylation of histone H3, Aurora B, and NuMa with increasing exposure time. HT-29 cells were treated with 30 nM OAT-449 or vincristine for the indicated times. The expression of P-histone H3 (Ser10), P-AuroraB (Thr232) and P-NuMa (Ser395) was determined by immunoblotting. (**a**) Effect of OAT-449 or vincristine on histone H3 phosphorylation in HT-29 cells after treatment for 1 h-18 h. (**b**) Effect of OAT-449 or vincristine on Aurora B phosphorylation in HT-29 cells after treatment for 1 h-18 h. (**c**) Effect of OAT-449 or vincristine on NuMa, histone H3 and Aurora B phosphorylation in HT-29 cells after treatment for 18 h (Lanes 1–2 represent biological replicates for each experimental condition). Densitometry of immunoblot data represents signal intensity relatively to β-tubulin (mean ± SEM). Data are representative of 3 or more independent experiments.

To analyze the molecular mechanism underlying mitotic catastrophe induced by both microtubule poisons in HT-29 cells, we performed Western blot analysis of the phosphorylation status of Cdk1, histone-H3, Aurora B and NuMa. In Figure 6, cells treated with OAT-449 for 18 h have reduced phosphorylation of Cdk1 (Thr15). Furthermore, Figure 7 shows that phosphorylation of Aurora B (Thr232), histone-H3 (Ser10) and NuMa (Ser395) is significantly increased compared to untreated cells. In all cases, we observed similar changes in phosphorylation upon exposure to treatment with either OAT-449 or vincristine.

### 3.7. OAT-449 or Vincristine Treated HT-29 Cells Arrested at G2/M Phase Have Increased Levels of p21, and This p21 Is Predominantly Cytoplasmic

**Figure 8:**
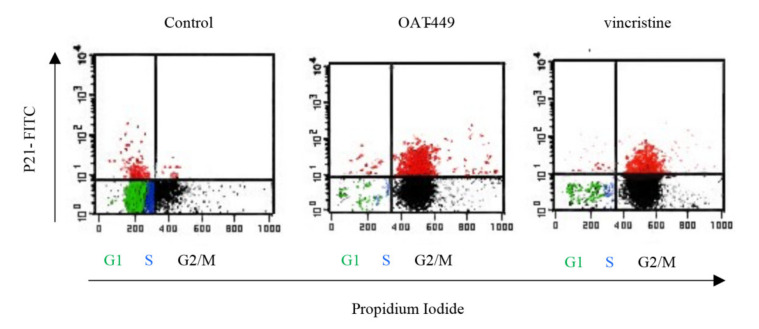
Increased expression of p21 in cells exhibiting G2/M phase arrest. HT-29 cells were treated for 24 h with 30 nM of OAT-449, vincristine or DMSO as a control. Cells were stained with anti-p21-FITC antibody and propidium iodide, with 10,000 cells analyzed per sample. Plots shown are representative of at least three independent experiments.

**Figure 9:**
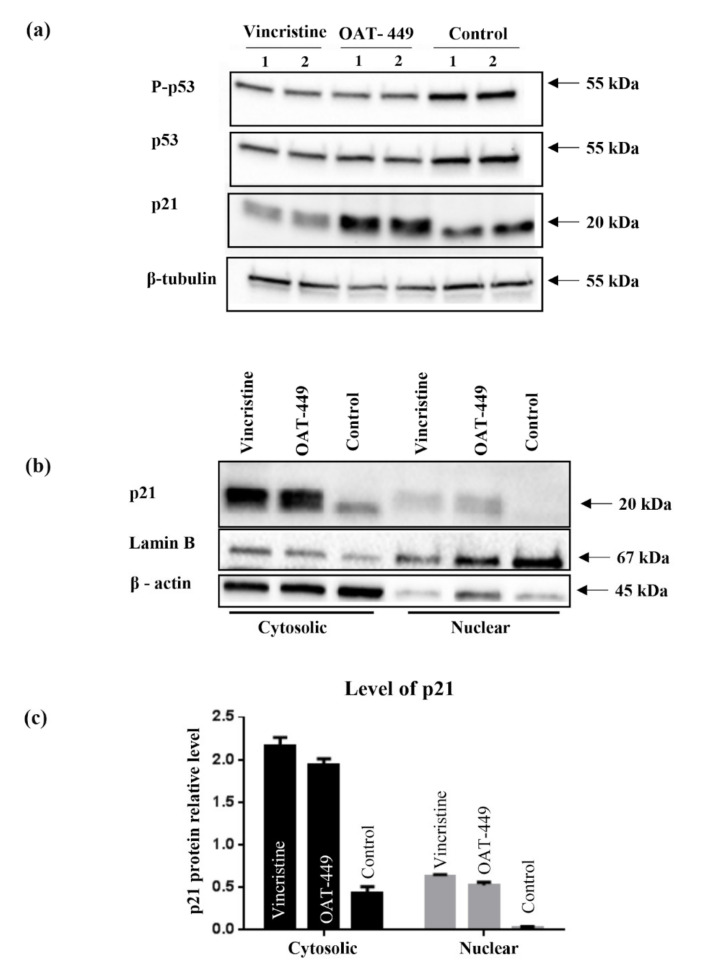
Correlation of p21 and p53 proteins expression level and their distribution between cytosolic and nuclear fractions after treatment with 30 nM OAT-449 or vincristine. (**a**) Immunoblot of p53/P-p53 (Ser15) and p21 proteins using whole cell lysate of HT-29 cells in basal condition and upon treatment with 30 nM OAT-449 or vincristine. (Lanes 1–2 represent biological replicates for each experimental condition). (**b**) Immunoblot of p21 protein in the cytoplasmic and nuclear fraction of HT-29 cells in basal condition and after 18 h treatment with 30 nM OAT-449 or vincristine. Lamin B and β-actin were used as a loading control for nuclear and cytoplasmic proteins. (**c**) Graph represents summary of densitometry of at least 3 independent western blot analyses (mean ± SD).

To search for the mechanism responsible for G2/M arrest and inhibition of apoptosis after OAT-449 or vincristine treatment, we focused on p21 protein, a member of the cyclin-dependent kinase inhibitors known as one of the key determinants of such cell fate programs as cell cycle progression, apoptosis or senescence. After OAT-449 treatment for 18 h, we observed an increase in p21 protein levels which clearly occurred in those cells that are arrested in the G2/M phase of the cell cycle (Figure 8). This suggests that p21 protein is responsible for the G2/M arrest. In addition to the nuclear function of p21 in cell cycle arrest, p21 located in the cytoplasm is known as an apoptosis inhibitor [23]. For example, cytoplasmic p21 has been reported to protect etoposide-induced apoptosis in leukemia cells [24]. As shown in Figure 9, in contrast with untreated control cells, both vincristine and OAT-449 exposure resulted in a large increase in the quantity of p21 protein. This is accompanied by only a small increase in p53 and phospho-p53, suggesting that the increase in p21 is p53-independent. Our data further indicate that the localization of p21 is heavily biased to the cytoplasm in the presence of OAT-449.

## 4. Discussion

Microtubule poisons, such as Vinca alkaloids, are crucial tools in the treatment of a variety of cancers, predominantly due to their ability to induce cell death at low concentrations, but the mechanisms of their activity remain only partially understood, along with their negative side effects. Therefore, it is vital that the cancer cell responses to these drugs are better elucidated and that newer compounds can be identified that have lower systemic toxicity and higher bioavailability whilst maintaining their efficacy.

After identifying OAT-449 as a good candidate molecule from a pre-screen of 20 novel molecular variations based on a common backbone [14], we found that OAT-449 is cytotoxic for a range of human cancer cell lines, including colorectal adenocarcinoma HT-29 and neuroblastoma SK-N-MC cells, at concentrations that are congruent with established treatments. OAT-449 anti-tumor activity was also found in HT-29 and SK-N-MC xenografts mouse models in vivo. Furthermore, we studied the molecular mechanism of OAT-449 cytotoxicity, and found that in HeLa and HT-29 cells, OAT-449 disrupts microtubule polymerization, in a manner similar to the Vinca alkaloid vincristine. Next, we analyzed in detail the cellular responses to OAT-449 compared with vincristine in HT-29 cells. After 24 h treatment, the interference of both OAT-449 and vincristine with the expansion of microtubules in HT-29 cells, as well as in HeLa cells, causing a blockade during the G2/M phase and mitotic catastrophe characterized by generation of multi-nucleated cells, indicating polyploidy and/or aneuploidy. Notably, at this time, the apoptotic cell death was blocked, and the extensive death of cells observed following mitotic catastrophe 24 h later, i.e., 72 h after OAT-449 treatment, was mostly necrotic. In fact, upon HT-29 cells’ treatment with OAT-449, we observed a small (about 8–10% above control) increase in the externalization of Annexin V, signaling membrane inversion, as an early apoptotic step, although in the absence of any activation of the execution machinery—caspase-3 and PARP. This hints that in a small number of cells, apoptosis may be proceeding through a caspase-3 independent pathway [25,26], but in the majority of cells the apoptosis was blocked. Additional support to this hypothesis was provided with both HT-29 and HeLa cell lines exhibiting minimal subG1 peaks following treatment with vincristine or OAT-449.

For the explanation of this observation, we focused on cellular p21 levels. In cancer cells, p21 can act either as a tumor suppressor or an oncogene, depending strongly on subcellular localization and the intracellular environment (reviewed in [27]). In the nucleus, p21 mainly inhibits cyclin-CDK complexes, inhibiting cell proliferation and arresting the cell cycle. The most well-known effect is p21-mediated arrest in G1 phase, but mounting evidence demonstrates that p21 can also trigger G2/M arrest. In contrast, p21 accumulation in the cytoplasm promotes cell cycle progression and causes the inhibition of apoptosis by binding to and inhibiting the activity of proteins of both receptor-mediated and mitochondrial apoptotic pathways, including procaspase-3, caspase-8, caspase-10, stress-activated protein kinases (SAPKs) and apoptosis signal-regulating kinase 1 (ASK1) (reviewed in [28]). Increased concentration of cytoplasmic p21 protein, probably mediated through an anti-apoptotic function, can also be a strong predictor of cancer prognosis [29,30]. In addition, one of the main determinants of the dual p21 functions in cancer cells is the status of tumor suppressor p53 protein, the transcriptional activator of p21. Where functional p53 is abundant, p53-dependent functions of p21 block tumorigenesis, while p21 promotes tumorigenesis in the absence of functional p53. However, several factors other than p53 can cause a p53-independent increase in p21 levels. Given the prominent role of p21 as a multitasking positive or negative regulator in various tumor cells, its function in response to cancer therapeutics, including vincristine, remains to be established [27].

We found that in HT-29 cells, OAT-449 as well as vincristine caused p21 accumulation at a lower level in the nucleus compared to the extensive accumulation in the cytoplasm, where p21 is known to inhibit apoptosis. Cytosolic accumulation of p21 and its anti-apoptotic activity has previously been described following treatment with paclitaxel [31], a stabilizing microtubule poison, after phosphorylation of Thr145 (the Akt1 phosphorylation site). In turn, some increase in p21 in the nuclei is consistent with the ability of p21 to arrest the cell cycle in the G2/M phase. This scenario is further supported by the finding that after the treatment with both microtubule poisons, the increase in p21 protein levels occurred in those cells which are arrested in the G2/M phase of the cell cycle (Figure 7). Thus, upon treatment of cancer cells with OAT-449, p21 seems to be responsible for G2/M growth arrest and at the same time for inhibition of apoptosis. As there was no equivalent impact of OAT-449 on p53, this p21 upregulation is mediated in a p53-independent manner [27,32]. This observation is consistent also with the genetic background of HT-29 cells where constitutive over-expression of mutated, inactive p53 may account for the lack of proper regulation of apoptosis [33].

In addition, as expected after observation of the G2/M phase arrest of HT-29 and other cell lines, we found perturbations in the phosphorylation/expression status of a number of proteins involved in cell cycle progression. There are two critical checkpoints at which the cell cycle may be arrested between G2 and return to G1, in order that cells can attempt to properly divide the chromosomes equally between daughter cells without any damage to the DNA molecules. The purpose of the G2 checkpoint is the detection of DNA damage prior to cell division. Progression of the cell cycle through this checkpoint is modulated via Cdk1-cyclin B1 activation, primarily by dephosphorylation of Cdk1 by its phosphatase CDC25C [34]. Following passage of the G2 checkpoint, active Cdk1-cyclinB1 translocates to the nucleus, where mitotic entry is promoted. In line with this, our experiments show that 18 h after treatment with OAT-449, Cdk1 was dephosphorylated and thus activated, and downstream substrates of active Cdk1-cyclin B1, Aurora B and histone H3, became phosphorylated. Exposure to OAT-449 or vincristine increased phospho-Aurora B and phospho-H3 in a dose-dependent manner, which is consistent with cells having progressed beyond the G2 checkpoint and having entered the M phase, during which is the spindle assembly checkpoint (SAC). The SAC is present to ensure that all chromosomes are properly attached to the spindle, along what will become the cleavage furrow, and that there is sufficient tension on the microtubules to pull the chromatids into their separate daughter cells before separation [35]. Autophosphorylation of Aurora B at Thr232 enables it to successfully bind to INCENP [36], making it highly probable that treatment with OAT-449 does not prevent Aurora B translocating to the mid-line in preparation for anaphase [37]. It has also been demonstrated that alterations in Aurora B expression can have disastrous consequences for cleavage furrow formation in excess [38]. Inhibition of Aurora B in the presence of microtubule poison paclitaxel [39] has been shown to contribute to mitotic slippage, ending in cellular senescence.

Our data show also that OAT-449 or vincristine treatment drives an increase in the phosphorylation of the nuclear mitotic apparatus (NuMA) protein. NuMA is a key structural nuclear protein whose presence and phosphorylation status vary throughout mitosis, with a proposed model transitioning from non-phosphorylated (nuclear), through minimally phosphorylated (during disassembly of the nucleus), ending in hyperphosphorylation (spindle assembly) [40]. NuMA is phosphorylated by a number of different kinases, and it has been shown that NuMA phosphorylation is required for attachment to, and assembly of, the mitotic spindle [41]. We found an increase in the presence of Ser395 phospho-NuMA, through which it regulates binding of 53BP1 to damaged DNA [42]. The mobility of 53BP1 within the nucleus is restricted by the binding of phospho-NuMA, which limits the binding of 53BP1 to sites of DNA damage. NuMa phosphorylation at Ser395 is also known to separate NuMA from 53BP1 [43], which further complicates the picture with respect to this interaction. Nevertheless, the observed alterations of the proteins regulating the cell cycle in the nucleus and the extensive phosphorylation of histone H3 strongly indicate that the microtubule disruption upon treatment with OAT-449 caused changes in the nucleus, which is known to represent a significant factor contributing to cellular stress responses.

Altogether, the data show that colorectal adenocarcinoma HT-29 cells treated with OAT-449 exhibit classical signaling scenario of activation of Cdk1, NuMa, Histone H3 and Aurora B within 18 h. This strongly suggests that the main mechanism by which both OAT-449 or vincristine cause mitotic catastrophe is upregulation of cell cycle progression, conflating it with failed passage of the spindle assembly checkpoint due to interference with microtubule polymerization and lack of tension between the microtubules and the mitotic spindle.

Overall, our study demonstrated a similar cellular mechanism of activity and anti-tumor efficacy of OAT-449 and vincristine in vitro and in vivo. The advantage in potential application of OAT-449 is linked to its low molecular weight and simplicity of OAT-449 structure. As a result, OAT-449 is easily soluble in aqueous solvents and can be readily synthesized in large quantities, lowering costs of drug production and increasing its broader availability. Moreover, OAT-449 can be relatively easily modified or further derivatized for targeted drug delivery, based, for example, on antibody-drug conjugates (ADC). An example of an ADC strategy with an antimitotic drug is monomethyl auristatin E (MMAE) [44]. Such approaches might aid in overcoming the problems with off-target effects of microtubule-targeting drugs and pave the way to make microtubule blockade more specific to tumor cells.

## 5. Conclusions

This study describes antitumor activity of a 2-aminoimidazoline derivative, OAT-449, a novel small-molecule inhibitor of microtubule polymerization, and its molecular mechanism of activity is similar to vincristine.

OAT-449, similarly to vincristine, proved to be capable of killing a range of cancer cells via potent destabilization of microtubules and to inhibit tumor growth in HT-29 and SK-N-MC xenotransplant mouse models. In HeLa or HT-29 cells, OAT-449 or vincristine treatment caused G2/M arrest and mitotic catastrophe followed by non-apoptotic cell death. In HT-29 cancer cells, mechanistically, both OAT-449 and vincristine are associated with the classical signaling scenario of activation of Cdk1, NuMa, Histone H3 and Aurora B, promoting cell cycle progression. This is, however, strongly counteracted by their interference with microtubule polymerization, resulting in a lack of tension between the microtubules and the mitotic spindle. This prevents passage of the spindle assembly checkpoint and failure to complete the M phase, leading to mitotic catastrophe. The study reveals also that the inhibition of microtubule polymerization causes a p53-independent increase in p21 protein levels in G2/M cell cycle phase, both in the nucleus and to a greater extent in the cytoplasm of HT-29 cells. While associated downstream and upstream signaling mechanisms need to be further studied, this upregulation is the first identified consistent mechanism for the two microtubule dissociating agents which can account for G2/M arrest and blocking apoptosis, determining in this way the necrotic cell death pathway following mitotic catastrophe. Besides this, OAT-449, due to its small size and high solubility in water, is a promising chemotherapeutic drug that could obviate some of the challenges associated with traditional drugs.

## Supplementary Materials

The following are available online at https://www.mdpi.com/2072-6694/12/8/2161/s1, Figure S1: In vitro tubulin binding and cell cycle.
