# Supplementary material for: A New Inhibitor of Tubulin Polymerization Kills Multiple Cancer Cell Types and Reveals p21-Mediated Mechanism Determining Cell Death after Mitotic Catastrophe
Source: Cancers (Basel). 2020 Aug 4;12(8):2161. doi: 10.3390/cancers12082161 (PMC7463620; doi:10.3390/cancers12082161)
Supplement: Supplementary file 1 [file cancers-12-02161-s001.pdf]

Supplementary Materials

A new inhibitor of tubulin polymerization kills multiple cancer cell types and reveals p21-mediated mechanism determining the cell death after mitotic catastrophe

Mykola Zdioruk, Andrew James Want, Anna Mietelska-Porowska, Katarzyna Laskowska-Kaszub, Joanna Wojsiat, Agata Klejman, Ewelina Użarowska, Paulina Koza, Sylwia Olejniczak, Stanisław Pikul, Witold Konopka, Jakub Golab and Urszula Wojda

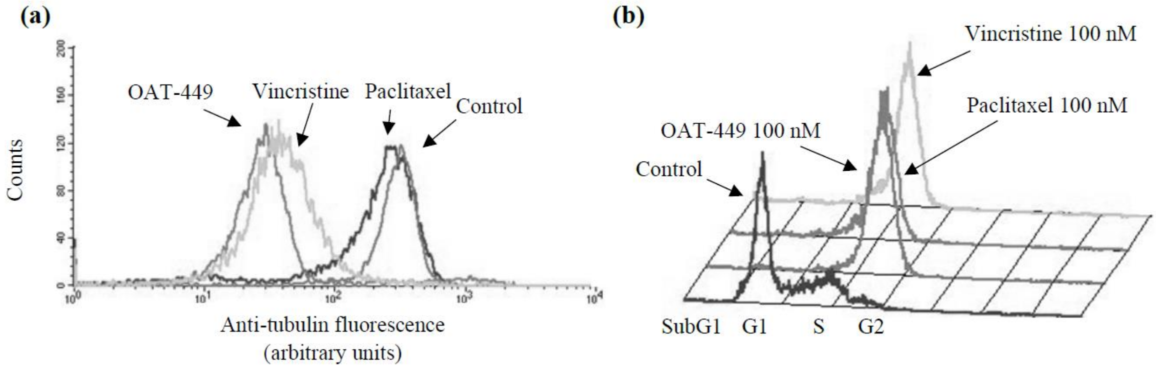

Figure S1. In vitro tubulin binding and cell cycle. Figure represents qualitative analysis of tubulin polymerization levels, with concurrent cell cycle analysis. HT-29 samples incubated for 18 h with 100 nM OAT-449, vincristine, paclitaxel or DMSO then stained with anti-β-tubulin antibody and PI and analyzed by flow cytometry. **(a)**-Representative plot showing fluorescence from tubulin binding, **(b)**-Cell cycle with different experimental conditions.

Below are uncropped figures for Western Blot figures in the main text.

(b)

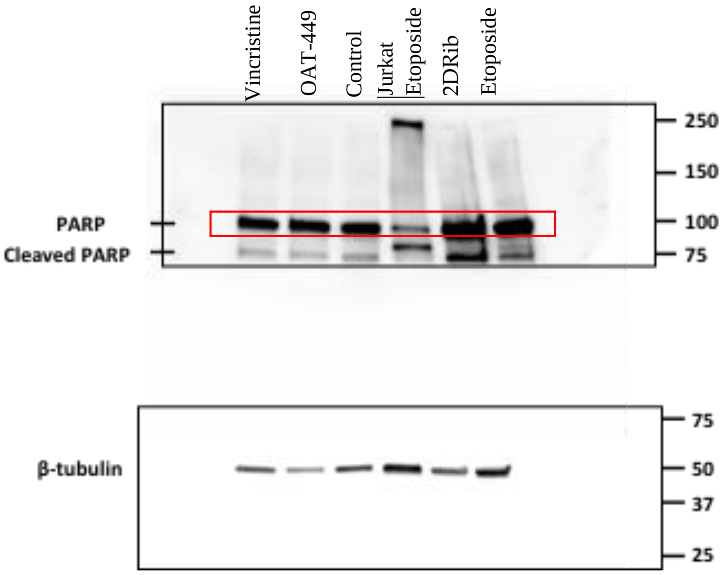

(c)

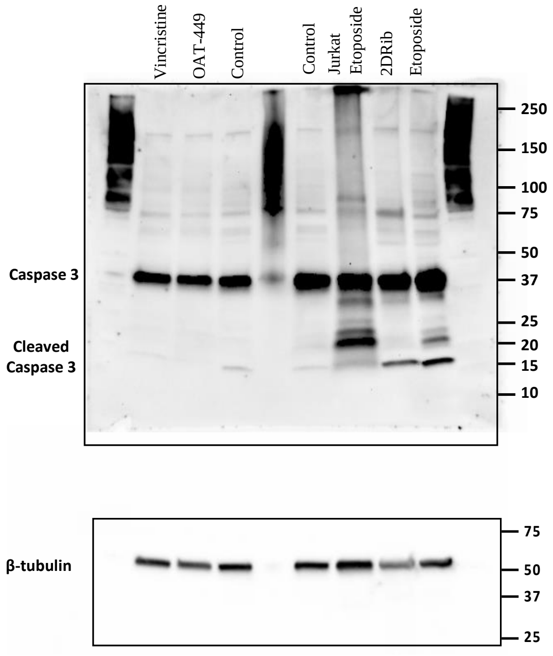

Detailed Information about Western Blot Analysis of Figure 4.

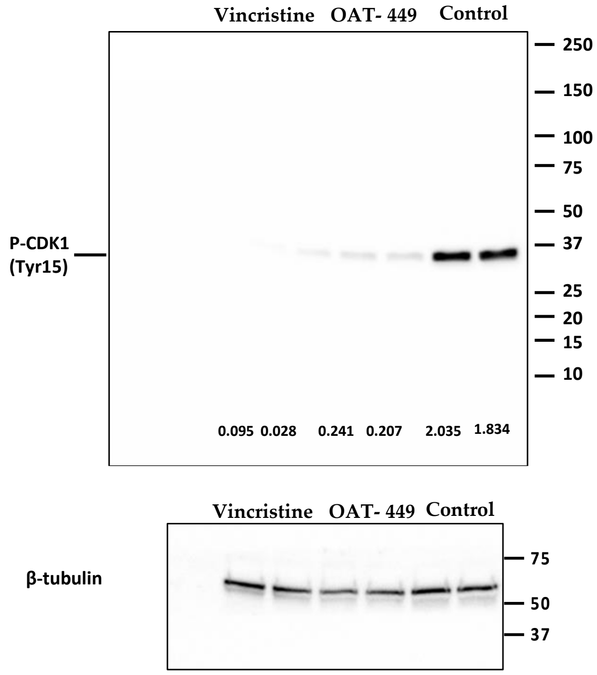

Detailed Information about Western Blot Analysis of Figure 6.

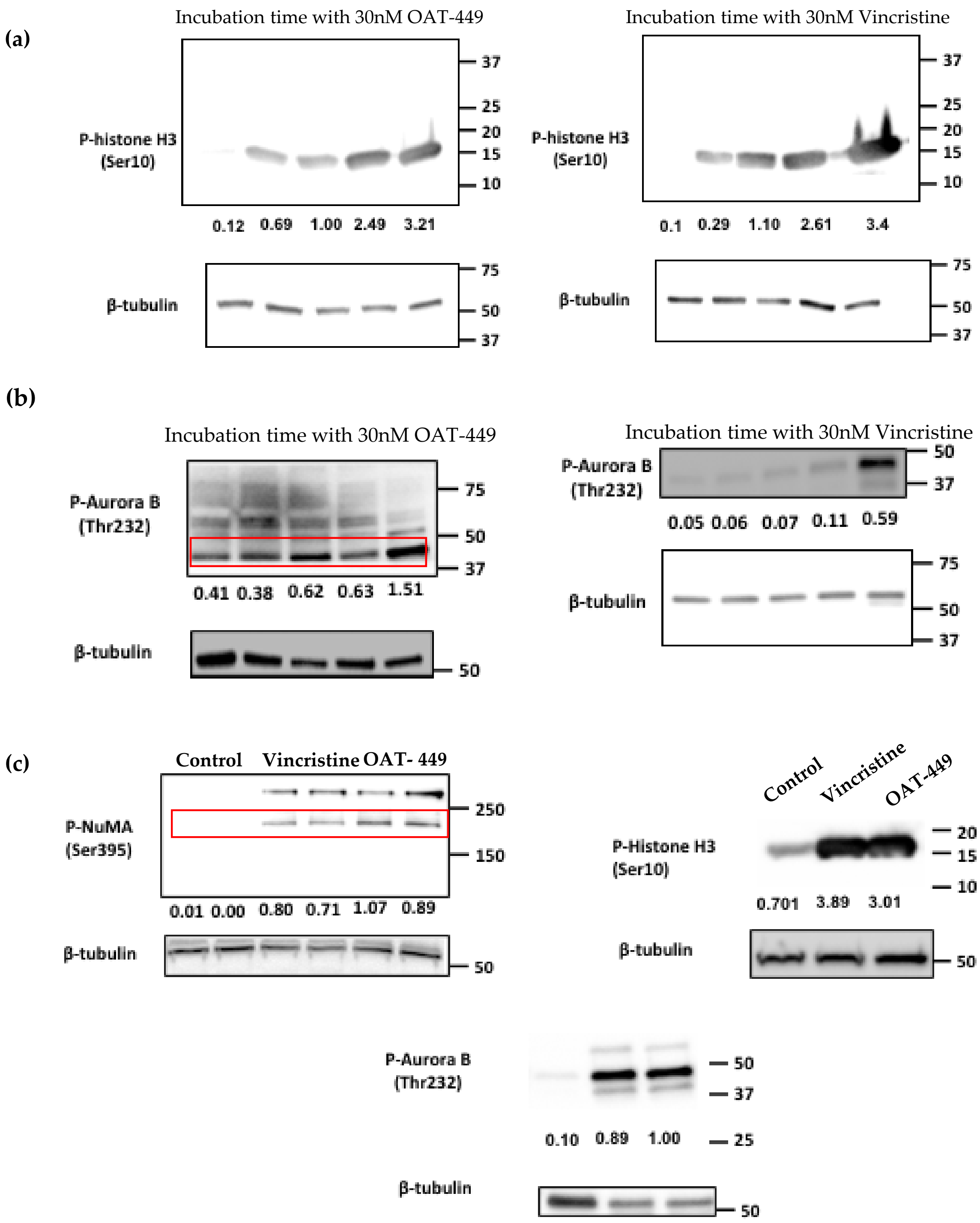

Detailed information about Western Blot Analysis of Figure 7.

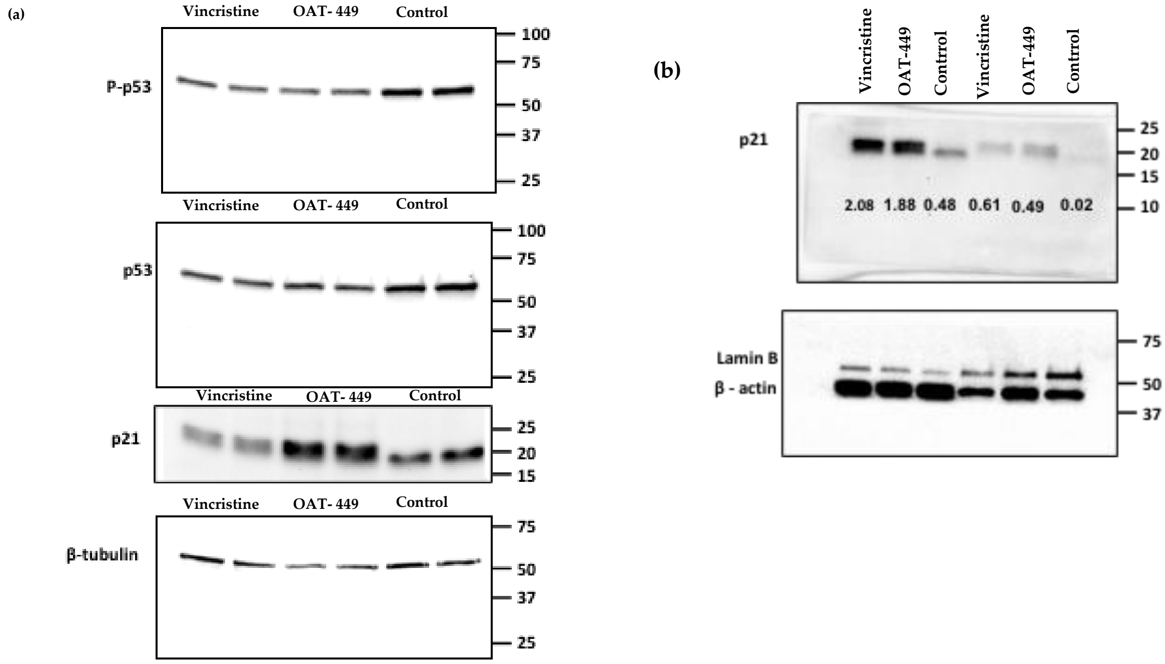

Detailed information about Western Blot Analysis of Figure 9.
